# Optic neuropathy and congenital glaucoma associated with probable Zika virus infection in Venezuelan patients

**DOI:** 10.1099/jmmcr.0.005145

**Published:** 2018-03-14

**Authors:** C. Gustavo De Moraes, Michele Pettito, Juan B. Yepez, Anavaj Sakuntabhai, Etienne Simon-Loriere, Mussaret B. Zaidi, Matthieu Prot, Claude Ruffie, Susan S. Kim, Rando Allikmets, Joseph D. Terwilliger, Joseph H. Lee, Gladys E. Maestre

**Affiliations:** ^1^​ Department of Ophthalmology, Columbia University Medical Center, New York, NY, USA; ^2^​ Clinica de Ojos de Maracaibo, Venezuela; ^3^​ Pasteur Institute, Functional Genetics of Infectious Diseases Unit, Paris, France; ^4^​ CNRS, URA 3012, Paris, France; ^5^​ Infectious Diseases Research Laboratory, Hospital General O'Horan, Merida, Mexico; ^6^​ Department of Epidemiology and Biostatistics, Michigan State University, East Lansing, MI, USA; ^7^​ Pasteur Institute, Viral Genomics and Vaccination Unit, Paris, France; ^8^​ CNRS, URA3015, Paris, France; ^9^​ In-patient Diabetes Unit, St. Peter’s Hospital, Albany, NY, USA; ^10^​ Departments of Psychiatry and Genetics and Development, Columbia University Medical Center, New York, NY, USA; ^11^​ Sergievsky Center, Columbia University Medical Center, New York, NY, USA; ^12^​ Division of Medical Genetics, New York State Psychiatric Institute, New York, NY, USA; ^13^​ Public Health Genomics Unit, National Institute for Health and Welfare, Helsinki, Finland; ^14^​ Taub Institute and Department of Epidemiology, Columbia University Medical Center, New York, NY, USA; ^15^​ Laboratory of Neuroscience, University of Zulia, Maracaibo, Venezuela; ^16^​ Department of Biomedical Sciences, Division of Neurosciences, University of Texas Rio Grande Valley School of Medicine, Brownsville, TX, USA; ^17^​ Department of Human Genetics, University of Texas Rio Grande Valley School of Medicine, Brownsville, TX, USA

**Keywords:** Zika virus, dengue, arbovirus, congenital glaucoma, uveitis, optic neuritis, vision loss, steroid treatment

## Abstract

**Introduction:**

Although the current Zika virus (ZIKV) epidemic is a major public health concern, most reports have focused on congenital ZIKV syndrome, its most devastating manifestation. Severe ocular complications associated with ZIKV infections and possible pathogenetic factors are rarely described. Here, we describe three Venezuelan patients who developed severe ocular manifestations following ZIKV infections. We also analyse their serological response to ZIKV and dengue virus (DENV).

**Case presentation:**

One adult with bilateral optic neuritis, a child of 4 years of age with retrobulbar uveitis and a newborn with bilateral congenital glaucoma had a recent history of an acute exanthematous infection consistent with ZIKV infection. The results of ELISA tests indicated that all patients were seropositive for ZIKV and four DENV serotypes.

**Conclusion:**

Patients with ZIKV infection can develop severe ocular complications. Anti-DENV antibodies from previous infections could play a role in the pathogenesis of these complications. Well-designed epidemiological studies are urgently needed to measure the risk of ZIKV ocular complications and confirm whether they are associated with the presence of anti-flaviviral antibodies.

## Introduction

Zika virus (ZIKV) is an arbovirus of the *Flaviviridae* family. It can be transmitted by the mosquito *Aedes aegypti* or through bodily fluids (e.g. blood, semen, saliva). Following the virus’ isolation in monkeys in the 1940s [1], it first appeared in humans in Africa in the 1960s [2]. In 2007, a large epidemic occurred in Micronesia [3]. Until then, ZIKV infection was considered benign, given that many infections presented as a mild form of dengue, with rash, fever, arthralgia and conjunctivitis, and a large proportion of the infections were asymptomatic [4, 5]. Serious outcomes were first identified during a 2013 Polynesian epidemic in which excess cases of Guillain–Barré syndrome (GBS), a peripheral neuropathic condition, were detected [6]; In 2014, GBS and microcephaly were reported in Brazil [7]. ZIKV outbreaks in North, Central and South America and the Caribbean followed, with significant regional differences in the scale, speed of transmission, and distribution of adverse outcomes [8]. These differences presumably resulted from a combination of interacting risk factors, such as demographic and health status of the local host populations, and characteristics of the vector and its surrounding environment. Other factors, such as health care accessibility, efficiency of reporting systems and health policies, could also affect the regional prevalence of ZIKV and its complications.

Severe ocular anomalies have been reported in infants with congenital ZIKV syndrome [9–12] and, more rarely, among adults [13–16]; however, none have been reported in children of 2 years of age or over. In consequence, the spectrum of severe ocular anomalies beyond congenital ZIKV syndrome is currently unknown, and our medical and public health practitioners lack sufficient knowledge about its diagnosis, clinical course and prevention. Optic neuropathy is defined as damage to the optic nerve (whether in the globe, orbit or intracranial space) clinically manifested by partial or total visual loss and/or abnormal findings in ophthalmological assessment. We report three cases of atypical, severe optic neuropathy, sight-threatening manifestations of ZIKV infection identified in 2016 in Maracaibo, Venezuela. Two patients, one adult and one child, were diagnosed with bilateral optic neuritis. A third patient, a newborn, had bilateral congenital glaucoma associated with probable vertical transmission of ZIKV.

## Case report

### Case 1

**Fig. 1.**
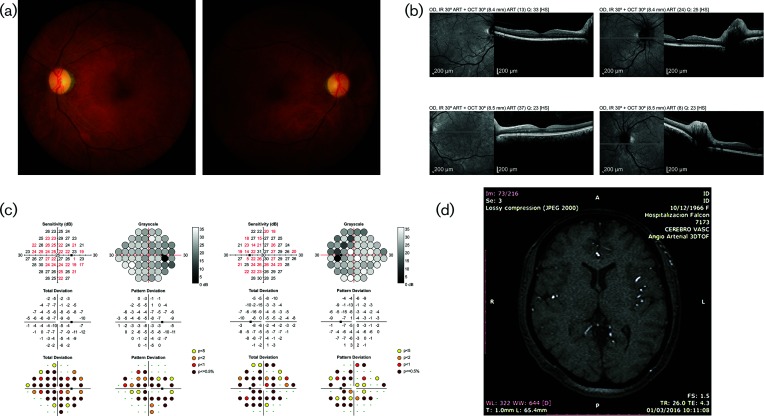
Patient described in Case 1. (a) Fundus photographs showing the optic nerve heads and maculas of the left and right eyes. (b) Optical coherence tomography (OCT) of the left and right eyes showing optic disc swelling; both macular scans are normal. (c) Visual field testing results showing diffuse contrast sensitivity loss. (d) Magnetic resonance imaging (MRI) of the brain showing no abnormalities.

A 49-year-old woman with no comorbidities was referred to the ophthalmology clinic for evaluation of sudden painless bilateral amaurosis. Nineteen days previously, she had been evaluated at an ambulatory care unit in Maracaibo, Venezuela, with complaints of headache, low fever and myalgia. Upon ophthalmological examination, the best-corrected visual acuity (BCVA) was OD 20/60, OS 20/40. There was mild conjunctival hyperaemia, no corneal abnormalities and no sign of anterior uveitis. Fundus examination (Fig. 1a) and optical coherence tomography (OCT, Fig. 1b) revealed bilateral swelling of the optic nerve head. Automated perimetry was performed to assess the visual field status. There was a diffuse loss of contrast sensitivity in both eyes, which did not respect the horizontal or vertical meridians (Fig. 1c). Magnetic resonance imaging (MRI) of the brain was unremarkable (Fig. 1d).

On the basis of the clinical manifestations and ancillary test results, the patient was diagnosed with bilateral optic neuritis of possible infectious or parainfectious origin. The absence of ocular pain, bilateral presentation and normal brain MRI made multiple sclerosis unlikely as the cause of neuritis. The patient reported no symptoms suggestive of arteritic ischaemic optic neuropathy, such as mandibular claudication or temporal headache. Nonarteritic ischaemic optic neuropathy was also deemed unlikely, given the absence of underlying vascular disease, hypertension, diabetes or hypercholesterolaemia.

The patient was treated with intravenous methylprednisolone (250 mg QID) for 3 days, followed by oral prednisolone (1 mg kg^−1^ day^−1^) for 11 days. The patient’s BCVA remained the same at the most recent ophthalmological examination, 20 months after the onset of symptoms. Fundus examination no longer revealed optic nerve head swelling, and the visual fields revealed mild improvement. The patient was seropositive for ZIKV and all four DENV serotypes (Table 1).

**Table 1.** Antibody titres to Zika virus (ZIKV) and Dengue virus (DENV) serotypes 1 to 4 in patients with severe ocular complications post-ZIKV infection

Patient	ZIKV IgG	DENV 1 IgG	DENV 2 IgG	DENV 3 IgG	DENV 4 IgG
Case 1	1785	288	226	276	312
Case 2	3320	1084	11521	6706	752
Case 3	836	1885	1374	1465	1424
Mother of Case 3	3463	4913	2729	3676	3014

### Case 2

**Fig. 2.**
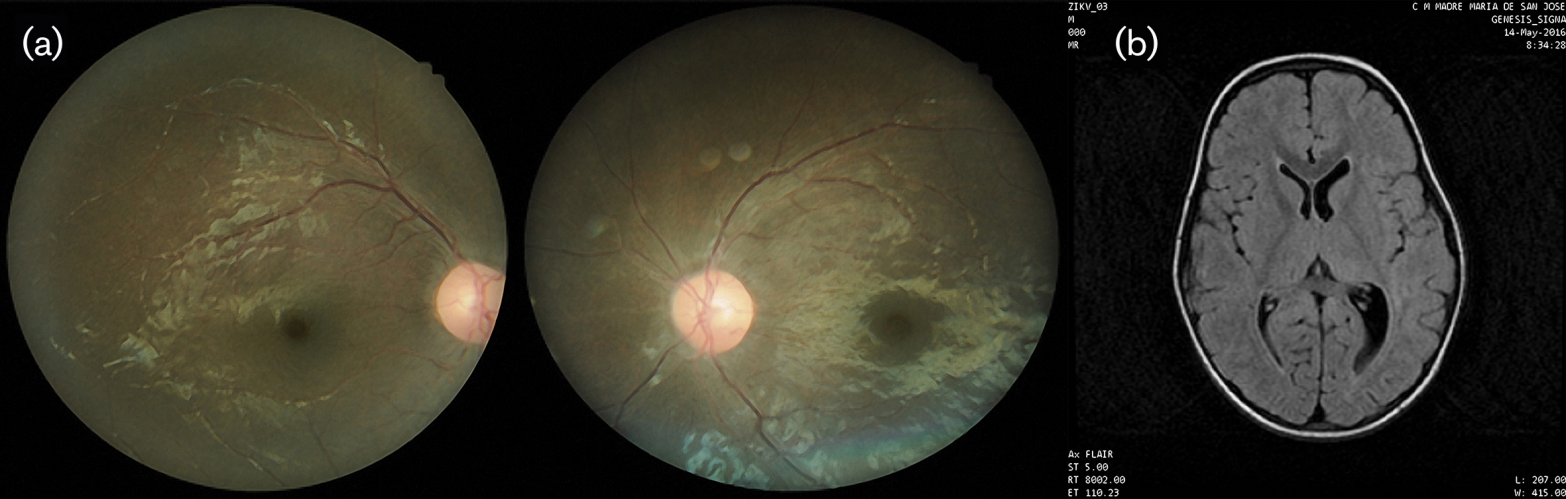
Patient described in Case 2. (a) Fundus examination revealed optic disc pallor and an edematous nerve fibre layer. Due to poor visual acuity, the patient was unable to perform visual field or optical coherence tomography (OCT) scans. (b) Magnetic resonance imaging (MRI) of the brain showing no abnormalities.

A previously healthy 4-year-old boy was referred to the ophthalmology clinic with sudden bilateral amaurosis. Two weeks prior to the onset of visual symptoms, the patient had a low fever, mild rash and myalgia, consistent with clinical manifestations of ZIKV infection. Ophthalmological evaluation showed that the patient’s BCVA was no light perception in either eye. Direct and consensual photomotor reflexes were hyporeactive. Anterior segment examination revealed no corneal abnormalities and no signs of uveitis. Fundus examination revealed optic disc pallor and an oedematous nerve fibre layer. The remaining fundus examination and MRI were unremarkable (Fig. 2). Due to poor visual acuity, the patient was unable to undergo OCT or visual field testing. No other ocular or systemic manifestations were observed.

The patient was also diagnosed with optic neuritis of possible infectious or parainfectious origin. He was treated with intravenous methylprednisolone for 3 days, followed by oral prednisolone for 11 days. At the last ophthalmological examination, 35 days after the onset of symptoms, his visual acuity had improved to light perception in both eyes. The patient was seropositive for ZIKV and all four serotypes of dengue (Table 1).

### Case 3

A 17-day-old infant was brought in for examination due to buphthalmia, epiphora, photophobia and blepharospasm [12]. Both eyes revealed cloudy corneas with diffuse oedema that precluded examination of the anterior chamber and fundoscopy. Intraocular pressure was 30 and 33 mm Hg in the right and left eye, respectively. Corneal diameter was 12.5 mm in each of the eyes. Notably, there were no signs of microcephaly, and the remaining systemic examination was unremarkable. The mother reported clinical symptoms compatible with ZIKV infection around the 25th week of pregnancy.

The patient was diagnosed with congenital glaucoma and underwent surgery in both eyes (360-degree ab interno trabeculotomy, combined with trabeculectomy). Postoperative intraocular pressure measurements were 15–20 mm Hg. Both the patient and her mother had IgG antibodies against ZIKV and all four dengue serotypes (Table 1). The patient received cornea transplants 20 months after the diagnosis.

### Assessment of humoral immunity

Sera from all three patients and the newborn’s mother were obtained when the patients arrived at our clinic for medical care. Antibody titres for ZIKV and DENV serotypes 1–4 were determined using an in-house ELISA assay. Serum samples were heat-inactivated for 30 min at 56 °C. Microtitre plates were coated with recombinant EDIII protein (either ZIKV or DENV 1–4) and incubated with serial dilutions of the test sera. Bound antibodies were revealed with an anti-human immunoglobulin G (IgG)-antibody horseradish peroxidase conjugate (Rockland) and 3,3′,5,5′–tetramethylbenzidine (TMB) substrate. The endpoint titre was calculated as the reciprocal of the highest dilution of individual serum, giving an absorbance of 0.5. All the samples were processed in the same batch. IgG titres to ZIKV and DENV 1–4 for each patient are shown in Table 1. All the sera were also tested for the presence of anti-ZIKV IgM and IgG antibodies, using a commercial anti-Zika antibody kit (EUROIMMUN, Lubeck, Germany). All patients were positive for anti-ZIKV IgG by both the in-house assays and the commercial kit, but none of the sera was positive for anti-ZIKV IgM antibodies. Neutralization assays were not performed.

## Discussion

We report two cases of optic neuropathy in an otherwise healthy adult and a child, both with probable recent ZIKV infection. Although we cannot conclusively demonstrate a causal relationship between ZIKV infection and optic neuritis due to the non-availability of acute samples, these cases indicate that the clinical manifestations of ZIKV infection could be more diverse and severe than previously assumed. Given that severe ocular complications resulting in permanent visual impairment [17, 18] have been associated with other arboviral infections, their presentation in ZIKV infection is not unexpected.

Initial reports of ocular manifestations of ZIKV infection in children with microcephaly [19] included focal pigment mottling and chorioretinal atrophy, primarily located in the posterior pole of the eye, especially the macular area. Such ocular manifestations are commonly seen in TORCH infections, which could reflect similar pathogenetic mechanisms among these different viral families [20, 21]. Subsequent reports on ocular pathology of ZIKV included cases of acute maculopathy [13, 14], uveitis [15, 16] and congenital glaucoma [22]. It is possible that ZIKV can cross the blood–brain barrier and lead to direct damage and an inflammatory response, even among healthy adults. This is more plausible among newborns given the immaturity of their nervous and immunological systems. The newborn we hereby describe could represent a spectrum of congenital manifestations of ZIKV vertical transmission, even in the absence of microcephaly [23, 24].

Current knowledge of the natural history of ZIKV is insufficient to identify who is at risk for severe or long-term adverse outcomes; therefore, it is difficult to determine why some ZIKV-infected individuals develop serious ocular complications and others do not. For example, we cannot explain geographical differences in the proportions and types of adverse outcomes [8] or variability in the clinical spectrum and symptomatic rates among infected individuals. The three cases reported herein exhibited IgG antibodies to all four dengue serotypes; in the case of the newborn, these are surely of maternal origin. While this demonstrates that dengue is hyperendemic in Venezuela, it appears to support the hypothesis that previous immunity to DENV can play a role in the pathogenesis of severe ZIKV infection [25]. However, because of the possibilities of (1) severe outcomes being related to previous ZIKV, DENV or both; and (2) cross-reaction between ZIKV and DENV, it is not possible to rule out prior dengue infection as a reason for the severe outcomes. A formal case–control study will be required to clarify whether prior or concurrent DENV infection or vaccination might contribute to severe ocular complications after ZIKV infection, or conversely, if previous exposure to ZIKV, with or without seropositivity to any of the DENV serotypes, is a risk factor for severe ocular outcomes. It is interesting to note that individuals who have been vaccinated against dengue can experience more severe symptoms and prognosis when exposed to a new strain [26]. This situation is believed to be mediated by antibody-dependent enhancement, in which specific antibodies to the previously infecting DENV serotype facilitate the entry of a newly infecting DENV serotype into host cells. The amino acid sequence of DENV and ZIKV differ by 41–46 %, and antibodies to these viruses have been shown to be cross-reactive [27].

The severe ocular complications associated with ZIKV infection are a major public health concern and warrant future studies. We recommend that all adults and children living in endemic areas who present with symptoms of ZIKV infection, as well as newborns whose mothers were infected during pregnancy, be carefully assessed to determine early signs of vision loss. Patients should be educated about the potential risk, to raise awareness and increase early detection, and undergo a complete ophthalmological examination if necessary. Patients with a history of ZIKV or DENV infection who present optic neuritis not explained by any other cause should be tested for antibodies against ZIKV, DENV 1–4 and possibly other arboviruses. Well-designed epidemiological studies are urgently needed to measure the risk of ZIKV ocular complications and to confirm whether they are associated with the presence of anti-flaviviral antibodies.
