# Corrigendum: Optic neuropathy and congenital glaucoma associated with probable Zika virus infection in Venezuelan patients

**DOI:** 10.1099/jmmcr.0.005161

**Published:** 2018-08-31

**Authors:** C. Gustavo De Moraes, Michele Pettito, Juan B. Yepez, Anavaj Sakuntabhai, Etienne Simon-Loriere, Mussaret B. Zaidi, Matthieu Prot, Claude Ruffie, Susan S. Kim, Rando Allikmets, Joseph D. Terwilliger, Joseph H. Lee, Gladys E. Maestre

**Affiliations:** ^1^​ Department of Ophthalmology, Columbia University Medical Center, New York, NY, USA; ^2^​ Clinica de Ojos de Maracaibo, Venezuela; ^3^​ Pasteur Institute, Functional Genetics of Infectious Diseases Unit, Paris, France; ^4^​ CNRS, URA 3012, Paris, France; ^5^​ Infectious Diseases Research Laboratory, Hospital General O'Horan, Merida, Mexico; ^6^​ Department of Epidemiology and Biostatistics, Michigan State University, East Lansing, MI, USA; ^7^​ Pasteur Institute, Viral Genomics and Vaccination Unit, Paris, France; ^8^​ CNRS, URA3015, Paris, France; ^9^​ In-patient Diabetes Unit, St. Peter’s Hospital, Albany, NY, USA; ^10^​ Departments of Psychiatry and Genetics and Development, Columbia University Medical Center, New York, NY, USA; ^11^​ Sergievsky Center, Columbia University Medical Center, New York, NY, USA; ^12^​ Division of Medical Genetics, New York State Psychiatric Institute, New York, NY, USA; ^13^​ Public Health Genomics Unit, National Institute for Health and Welfare, Helsinki, Finland; ^14^​ Taub Institute and Department of Epidemiology, Columbia University Medical Center, New York, NY, USA; ^15^​ Laboratory of Neuroscience, University of Zulia, Maracaibo, Venezuela; ^16^​ Department of Biomedical Sciences, Division of Neurosciences, University of Texas Rio Grande Valley School of Medicine, Brownsville, TX, USA; ^17^​ Department of Human Genetics, University of Texas Rio Grande Valley School of Medicine, Brownsville, TX, USA

**Keywords:** Corrigendum, Zika virus, dengue, arbovirus, congenital glaucoma, uveitis, optic neuritis, vision loss, steroid treatment

There was an error in the text in the published article.

The abstract states ‘a child of 4 years of age with retrobulbar uveitis’, this should be ‘a child of 4 years of age with retrobulbar neuritis’.

The author apologizes for any inconvenience caused.

